# Tularemia in a Park, Philadelphia, Pennsylvania

**DOI:** 10.3201/eid1409.071690

**Published:** 2008-09

**Authors:** Julie R. Sinclair, Alisa Newton, Keith Hinshaw, George Fraser, Patrina Ross, Esther Chernak, Caroline Johnson, Nancy Warren

**Affiliations:** Centers for Disease Control and Prevention, Atlanta, Georgia, USA (J.R. Sinclair); Philadelphia Zoo, Philadelphia, Pennsylvania, USA (A. Newton, K. Hinshaw); Pennsylvania Bureau of Laboratories, Lionville, Pennsylvania, USA (G. Fraser, N. Warren); Philadelphia Department of Public Health, Philadelphia (P. Ross, E. Chernak, C. Johnson)

**Keywords:** tularemia, Francisella tularensis, zoonosis, biological weapon, biomonitoring, letter

**To the Editor:** Tularemia is a bacterial zoonosis caused by the gram-negative, nonmotile coccobacillus *Francisella tularensis,* which is endemic in lagomorphs in North America (*1*,*2*). Tularemia is considered a possible biological weapon of terrorism (Centers for Disease Control and Prevention [CDC] category A) because of its high infectivity, ease of dissemination, and considerable ability to cause illness and death in humans (*3*). The BioWatch Program monitors the environment in urban areas throughout the United States for *F. tularensis* and other potential bioterrorism agents. The epidemiology of many of these pathogens in urban ecosystems is not well understood; reservoirs may not be known or suspected, which leads to an inability to differentiate natural infection from a bioterrorism event. We describe a cluster of tularemia infections (in the absence of identified human illness or environmental detection) in feral rabbits found in a 0.5-km^2^ area of a large city park in Philadelphia, Pennsylvania, USA.

During the spring and summer of 2006, a total of 14 eastern cottontail rabbits (*Sylvilagus floridanus*) and 2 woodchucks (*Marmota monax*) were found dead or trapped and euthanized (2 rabbits only) at a zoological park. The animals were necropsied, and specimens of liver and spleen were sent to the Pennsylvania Bureau of Laboratories (BOL) for *F. tularensis* culture and PCR. Two years earlier, in the spring of 2004, a single rabbit found dead at this same location had tested positive for *F. tularensis*; PCR and culture identified the organism in liver and spleen. Of the 14 rabbits submitted in 2006 for *F. tularensis* testing, 6 were positive (collection dates ranged from March through August). Five of these were positive by PCR and culture, and 1 was positive by PCR alone; *F. tularensis* was identified only in animals found dead. The 2 woodchucks tested negative by PCR and culture. The 2004 isolate and 2006 isolates were identified by CDC as type A *F. tularensis* and were found genetically identical by pulsed-field gel electrophoresis.

These additional 2006 positive findings triggered efforts to use available resources to identify other tularemia sources: the Philadelphia Department of Public Health (PDPH) heightened surveillance for tularemia by requesting that other city agencies and wildlife rehabilitation centers report and submit for testing any mammals found dead from unknown causes. (City agencies reported a few larger mammals, e.g., groundhogs and raccoons, dead from trauma; these animals were not tested.) The zoological park continued routine illness monitoring of collection animals, animals on grounds, and staff. In addition, during October 2006 and March 2007, the PDPH collected ticks on the outskirts of a heavily wooded area with frequent foot traffic ≈1.5 miles from the site where the rabbits were found dead. (The specific tick collection method involved dragging a white cotton bath towel along the edge of a wooded area; this activity took place during the hours of 10:00 AM–2:00 PM Other tick-dragging attempts during August 2007, on the outskirts of a heavily wooded area ≈0.5 miles away that was accessible to foot traffic but across the river from the zoological park, yielded no results.) A total of ≈30 deer ticks (*Ixodes scapularis*, which are not a known vector for tularemia) were collected each month; no other species were identified. These tick specimens were submitted to BOL for *F. tularensis* testing by PCR and culture. During November and December 2006, 5 crayfish (*Procambarus acutus acutus*, cited as a possible reservoir for type B tularemia by Anda et al.) (*4*), were trapped from a pond near the site where the rabbits were found dead and submitted to BOL for *F. tularensis* testing by PCR and culture. None of these readily available surveillance activities resulted in identifying tularemia except in the rabbits found dead in the zoological park. Additionally, no cases of human tularemia were reported to PDPH during this period, despite distribution of a health alert to medical providers to heighten clinical suspicion for the disease. Furthermore, the organism was not detected by routine environmental monitoring of air samples by the city’s BioWatch sensors.

Even though this limited investigation failed to identify additional *F. tularensis* infections in humans and in any of the animals and ticks tested, the cluster of infections in rabbits in Philadelphia indicates that *F. tularensis* is present in the environment in sufficient numbers to cause a noteworthy die-off of animals (i.e., 6 rabbits in a 0.5-square-mile area over a 5-month period). Environmental biomonitors in other metropolitan areas have been triggered by reported detection of tularemia on at least 2 occasions in the past 5 years—Houston in 2003 and the Washington, DC, National Mall in 2005 (*5*).

This investigation underscores that *F. tularensis* identification in the environment requires a systematic approach beyond environmental biomonitoring, random convenience sampling, and increased passive surveillance for human cases. Standard methods such as serologic studies of wildlife may not be available to resource-limited urban institutions. Possible strategies such as the collection of ticks, specifically the American dog tick, *Dermacentor variabilis* (a known vector for tularemia), from animals upon entry into urban animal shelters and mapping of areas where the animals were found need to be considered if resources are limited. Additional research is necessary to understand the occurrence of disease caused by *F. tularensis* in humans and animals, especially in urban environments (*6*).
